# 

**Published:** 1860-01

**Authors:** 


NORTH AMERICAN

### MEDICO-CHIRURGICAL REVIEW.

Vol. IV.] JANUARY, 1860. [No. 1.

### CONTENTS.

### ANALYTICAL AND CRITICAL REVIEWS.

ART. PAGE

I.—A Practical Treatise on the Diagnosis, Pathology, and Treatment of Diseases of the Heart. By AUSTIN FLINT, M.D.... 1

II.—Notes on the Surgery of the War in the Crimea; with Remarks on the Treatment of Gunshot Wounds. By GEORGE H. B. MACLEOD, M.D.. 30

III.—A Practical Treatise on Enteric Fever; its Diagnosis and Treatment. By JAMES E. REEVES, M.D.... 57

IV.—An Introduction to Practical Pharmacy; with many Formulæ and Prescriptions. By EDWARD PARRISH, Graduate in Pharmacy, etc.... 69

V.—The Obstetric Catechism; containing 2347 Questions and Answers on Obstetrics Proper. By JOSEPH WARRINGTON, M.D.... 70

VI.—Lectures on Surgical Pathology. By JAMES PAGET, F.R.S.... 70

VII.—The Action of Medicines on the System. By FREDERICK WILLIAM HEADLAND, M.D.... 71

VIII.—The Physician's Hand-book of Practice, for 1860. By WILLIAM ELMER, M.D., and LEWIS ELSBERG, M.D.... 71

IX.—The Physician's Visiting List, Diary, etc., for 1860.... 72

X.—The Surgery of Western Nations. By H. HOBSON, M.D.... 72

XI.—Description of a Deformed, Fragmentary Human Skull. By J. AITKEN MEIGS, M.D.... 73

### ORIGINAL COMMUNICATIONS.

I.—Cases from the Surgical Clinic of the Jefferson Medical College.—Service of PROF. GROSS. By EMIL FISCHER, M.D.... 73

II.—A Case of Degenerated Bronchocele of Enormous Size, cured by Medical and Surgical Treatment. By O. B. KNODE, M.D.... 88

III.—Treatment of Stomatitis Materna by the Syrup of the Phosphates. By E. J. FOUNTAIN, M.D.... 89

IV.—Spontaneous Amputation below the Knee. By JOHN G. KERR, M.D.... 91

V.—A Case of Inversion of the Uterus Successfully Reduced. By WM. IRVIN, M.D.... 93

VI.—Proceedings of the Pathological Society of Philadelphia.... 93

### BI-MONTHLY ABSTRACTS.

SURGERY. BY S. W. GROSS, M.D.

On Recto-Vesical Lithotomy.—Case of Large Vesical Calculus.—Maisonneuve's Operation for the Removal of Naso-pharyngeal Fibrous Polyps.—Ligature of the Common Iliac Artery, for Aneurism.—Amputation at the Hip-Joint.—Inguino-femoral Aneurism cured by Pressure on the External Iliac.—Remarks upon a Rare Variety of Tumor of the Scrotal Coverings.—On the Treatment of Epithelial Cancers by the Actual Cautery.—Ovariotomy in Ohio.—New Operation for the Relief of the Pain in Acute Glaucoma.... 121

PATHOLOGY AND PRACTICE OF MEDICINE. BY RICHARD J. DUNGLISON, M.D.

Movable Kidney, in connection with Spinal Disease.—Albuminuria in one Kidney, Saccharine Urine in the other.—Fatal Case of Contracted Bright's

PAGE

Kidney, with Pericarditis.—Total Suppression of Urine.—Interesting Cardiac Affections.—Rupture of the Right Auricle of the Heart.—Aneurism of the Abdominal Aorta arrested in its Progress.—Transitory Homicidal Mania.—Rapidly Fatal Anæmia.—Appendix Cæci discharged from the Bowels........ 129

OBSTETRICS, AND DISEASES OF WOMEN AND CHILDREN. BY WM. B. ATKINSON, M.D.

The Sickness of Pregnancy.—Uva Ursi as a Parturifaciant.—Records of Midwifery Practice.—The Treatment of Menorrhagia, etc., by Arsenic.—Chronic Inversion of the Uterus.—Treatment of Pelvi-Peritonitis.—Hydatidiform, or Vesicular Mole; its Nature, etc.—A Weeping Leg.—New Mode of Resuscitating Still-born Children.—On the Suction Method of Rearing Infants........ 136

PHYSIOLOGY. BY S. WEIR MITCHELL, M.D.

Action of Salts upon the Red Corpuscles of the Blood.—Normal Presence of Alcohol in the Blood.—Experimental Researches on the Influence of Light, etc. on the Iris of Vertebrate Animals.—On the Substance called Animal Starch.—The Capillaries of the Brain.—Coagulation of the Blood.. 143

MATERIA MEDICA AND THERAPEUTICS; MEDICAL CHEMISTRY; TOXICOLOGY AND LEGAL MEDICINE. BY EMIL FISCHER, M.D.

*Materia Medica and Therapeutics:* Comparative Researches on Oleum Morrhuæ, Rajæ, and Squali.—On different Species of Helleborus.—On Ranunculus Ficaria.—Glycerin; its Applications in Surgery and Medicine.—On Ozonized Oils.—Substitute for Fowler's Solution.—Creosote in Cough with Purulent Expectoration.—Muriate of Gold and Belladonna in Affections of the Stomach.—Chloroform in Hooping-cough.—Leaves of the Rhamnus Alaternus as an Antigalactic.—Digataline in Puerperal Fever.—Arsenious Acid in Chorea.—Hæmatic Capsules.—Glycerin Ointment for the Itch.—Bromine in Pseudo-membranous Affections........ 150

*Medical Chemistry:* On the Tests for Detecting Sugar in the Urine.—On the Green or Blue Color of Pus.—Researches on the Sugar found in the Glucogenous Matter of the Liver........ 161

*Toxicology and Legal Medicine:* Blood-Crystals, and their Judicial Medical Importance.—Case of Rape committed on a Child.—Physiological and Toxicological Properties of Tanghinia Venenifera.—Effect of Inhalation of Arseniuretted Hydrogen........ 163

BI-MONTHLY PERISCOPE.

SURGERY.

Tracheotomy in Croup.—Comments on the Series of Tracheotomy for Laryngeal Affections.—Sulphuric Ether as an Anæsthetic, its first use in Mass. General Hospital.—Extraction of Cataract by Linear Incision.—Needle for the Wire Suture........ 165

PRACTICE OF MEDICINE.

The Pathology and Treatment of Gall-Stones........ 177

EDITORS' TABLE.—The Gold-headed Cane.—Medical Students in Philadelphia.—Chicago Medical Examiner.—Professor Virchow.—Chicago College of Pharmacy.—Maine Medical School.—Philadelphia Lunatic Asylum, Blockley.—New York College of Veterinary Surgery.—Army and Navy Dentists.—American Medical Association.—Prize Questions.—Exodus of Southern Medical Students.—A Discourse Commemorative of the Life of Prof. T. D. Mütter, M.D., LL.D........ 182

NECROLOGICAL NOTICES.—Dr. J. P. Barret.—Charles Gardiner Guthrie.—Dr. Thomas Nuttall.—Dr. Gillette.—Baron Philippe Boyer.—Dr. Henfrey.—Dr. George Regnoldi.—Dr. Francis Barker........ 191

BI-MONTHLY BIBLIOGRAPHICAL RECORD........ 192

